# Non-Coding RNAs and Cancer

**DOI:** 10.3390/ijms140817085

**Published:** 2013-08-19

**Authors:** Federica Calore, Francesca Lovat, Michela Garofalo

**Affiliations:** Department of Molecular Virology, Immunology and Medical Genetics, Comprehensive Cancer Center, Ohio State University, Columbus, OH 43210, USA; E-Mails: federica.calore@osumc.edu (F.C.); francesca.lovat@osumc.edu (F.L.)

**Keywords:** small non-coding RNAs, long non-coding RNAs, cancer

## Abstract

The discovery of the biological relevance of non-coding RNA (ncRNAs) molecules represents one of the most significant advances in contemporary molecular biology. Expression profiling of human tumors, based on the expression of miRNAs and other short or long ncRNAs, has identified signatures associated with diagnosis, staging, progression, prognosis, and response to treatment. In this review we will discuss the recent remarkable advancement in the understanding the biological functions of human ncRNAs in cancer, the mechanisms of expression and the therapeutic potential.

## 1. Introduction

The human genome sequencing performed by the International Human Genome Sequencing Consortium revealed that the number of protein-coding genes corresponded only to 20–25,000 [1]. While, at first, it was common belief that the remaining, bigger portion of the human genome was not functional and considered as “junk DNA”, several studies based on advanced technologies such as tiling arrays and RNA deep sequencing have recently pointed out the identification of thousands of RNA transcripts not derived from known genes and not encoding for a protein [2,3]. These molecules have been classified as non-coding RNAs (ncRNAs).

NcRNAs could be grouped into two major classes based on the transcript size: small ncRNAs less than 200 bp, such as piRNAs (Piwi-associated RNAs), miRNAs (microRNAs), and snoRNAs (small nucleolar RNAs), and long ncRNAs (lncRNAs), greater than 200 bp. Each of these classes can be further divided, whereas novel subclasses are still being discovered and characterized. All these ncRNAs form huge molecular networks and play a central role in regulating cellular activities in Eukaryotes. The alteration and dysregulation of several ncRNA has been reported in various human diseases, including cancer, providing evidence for targeting these molecules as anticancer agents. Here, we will summarize the current knowledge about regulatory functions of ncRNAs, with special emphasis on their effects in cancer formation and progression.

## 2. piRNAs

**Figure 1:**
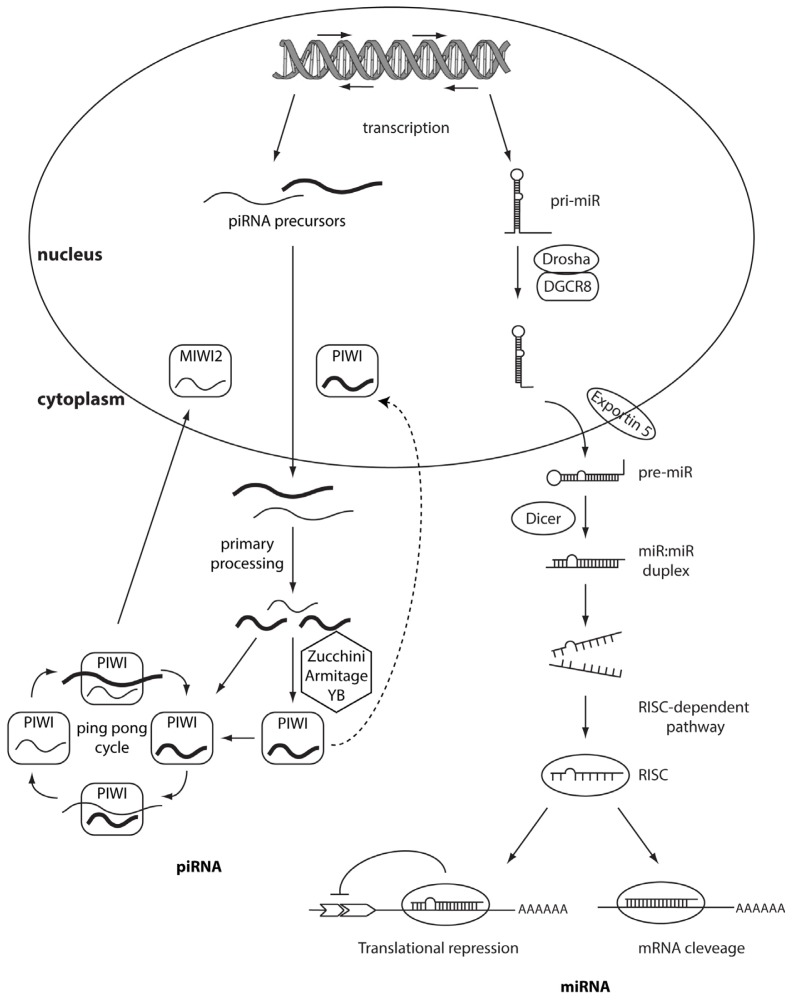
PiRNAs and microRNAs biogenesis. On the left, piRNAs biogenesis. PiRNAs are processed from single-stranded RNA precursors. The biogenesis of piRNAs could be divided in two main pathways: primary processing and ping-pong amplification cycle. MIWI2, a PIWI protein, translocates processed piRNAs into the nucleus, where they block the transcription of the TE (trasposon elements). On the right, miRNA biogenesis. Primary transcripts (pri-miRs) are transcribed by the RNA polymerase II. In the nucleus pri-miRs are processed by Drosha-DGCR8 into pre-miRs of ~60–70 nt. The produced pre-miRNAs are exported by the Exportin 5 to the cytoplasm where they are processed in ~18–22-nucleotide miRNA duplexes by the cytoplasmic RNase III Dicer. Normally, one strand of this duplex is degraded, whereas the other strand accumulates as a mature miRNA. From the miRNA-miRNA duplex, only the miRNA enters preferentially in the protein effector complex, formed by the RNA-induced silencing complex (RISC) and miRgonaute. Perfect or nearly perfect complementarities between miRNA and its target 3′ UTR induce RISC to cleave the target mRNA, whereas imperfect base matching induces mainly translational silencing of the target.

PIWI-family proteins and their associated small RNAs (piRNAs) provide an essential protection for germ-cell genomes against the activity of transponsable elements (TE). They help to maintain genome integrity, silencing TE [4] and this role is highly conserved across animal species. Unlike the other classes of small noncoding RNAs, which are 24–32 nt in length, they are generated from single-stranded RNA precursors through a Dicer-independent mechanism [5–7]. PiRNAs associate with PIWI proteins, which are germline-specific members of the Argonaute protein family, while siRNAs and miRNAs associate with ubiquitously expressed AGO subfamily members. The PIWI protein family is highly conserved across a variety of species and organisms. MIWI, MILI, and MIWI2 (Piwil4) are the three mouse PIWI proteins [8–10], whereas PIWIL1/HIWI, PIWIL2/HILI, PIWIL3, and PIWIL4/HIWI2 are the four PIWI proteins expressed in humans [11]. PIWI mutations in mice, Drosophila, and zebrafish, result in consistent defects in spermatogenic cells, demonstrating the essential role of PIWI proteins in germline development [12–15]. PiRNAs are more expressed in testes than other small noncoding RNAs [16–19] and are involved in spermatogenesis by regulating meiosis and/or suppressing TE. Hundreds of thousands of different piRNA species have been found in mammals [20], with no clear secondary structure motifs but with a common bias 5′ uridine. At 3′ termini piRNAs present a 2′-*O*-methylation, a process mediated by methyltransferase HEN1, which is associated with PIWI proteins [21,22]. This modification protects piRNA from 3′→5′ exonucleases activity, suggesting an increase of their stability [23]. PiRNAs are not distributed across the whole genome but they are clustered in few hundred genomic loci called piRNA clusters [6]. The biogenesis of piRNA could be divided into two main pathways: primary processing and ping-pong amplification cycle (Figure 1). First, piRNA clusters are transcribed in both directions and provide a pool of fragmented primary piRNAs. Primary piRNA transcripts are exported to the cytoplasm where numerous factors (*i.e*., Zucchini, Armitage and YB) participate in piRNA processing and loading onto PIWI proteins [24,25]. Piwi-piRNA complexes are transported into the nucleus, where they inhibit transcription of TE [26,27]. This first process (primary processing) is similar between germline and somatic cells. The next phase, the ping-pong amplification, is restricted to germline cells and requires slicer activity of PIWI proteins [25,26]. Recently, few works have demonstrated, by deep sequencing, that piRNA population is present in many more cell types than germline cells. For example, Lee *et al.* indentified the presence of a limited set of piRNAs in the mouse hippocampus. The most up-regulated, *DQ541777,* controls spine shape [28]. Moreover, another study described piRNA expression in more than 130 fruit fly, mouse, and rhesus macaque samples. Further, in mouse pancreas and macaque epididymis, piRNA are abundant as much as piRNA abundance in the germline [29]. An emerging number of studies highlights the role of piRNAs or PIWI proteins in the regulation of tumorigenesis. Indeed, piRNAs have been described in HeLa cells [30] and gastric, colon, lung, and breast cancer tissues [31]. These discoveries should not be surprising considering that cancer cells and germ cells share common features such as rapid proliferation and potentially infinite self-renewal. The first evidence of the role of piRNAs in cancer is described by Qiao *et al.* Hiwi, a Piwi family member, is over-expressed in seminomas but not in nonseminomas or in somatic tumors of the adult testis [32]. Moreover, HIWI over-expression has been also shown in cervical, pancreatic, colorectal, endometrial, esophageal, liver cancer, and gliomas [33–39]. Recently, Cheng and colleagues demonstrated that the expression of *piR-651* in gastric, colon, lung, and breast cancer tissues was higher compared to normal adjacent tissues. The levels of *piR-651* were associated with tumor-node-metastasis (TNM) stages. Inhibition of *piR-651* caused the arrest of gastric cancer cells at the G_2_/M phase [31]; therefore this pi-RNA shows an oncogenic role and plays a crucial function in carcinogenesis. Another study demonstrated the down-regulation of *piR-823* in gastric cancer tissues compared to normal tissues suggesting its potential tumor suppressive role [40]. *In vivo* studies showed that the over-expression of *piR-823* significantly inhibited tumor growth in a dose-dependent manner. Moreover, *piR-823* was significantly lower in peripheral blood of gastric cancer patients compared to healthy controls. The levels of *piR-823* were positively associated with TNM stages and distant metastasis, suggesting that *piR-823* should be tested as a biomarker for detecting circulating gastric cancer cells in the blood [41]. All these data may suggest an important role of the axis PIWI and PIWI-associated RNAs going beyond the regulation of the genome in germline tissues and more studies are needed in order to investigate their specific role in tumorigenesis.

## 3. MicroRNAs

In 1993, Victor Ambros and colleagues discovered a gene, *lin-4*, that affected the development of *Caenorhabditis elegans* and found that its product was a small nonprotein-coding RNA [42]. The number of known small RNAs in different organisms such as *Caenorhabditis elegans*, *Drosophila melanogaster*, plants, and mammals, including humans, has since expanded substantially. MicroRNAs (miRNAs) are 19- to 24- nucleotide non-coding RNA molecules that regulate the expression of target mRNAs both at the transcriptional and translational level [43,44]. While in plants such regulation occurs through perfect base-pairing, usually in the 3′ untranslated region (UTR) of the targeted mRNA, in mammals the base-pairing is only partial [45,46].

Each member of this large family of non-coding RNAs can have hundreds of different targets and nearly 30% of the genes are regulated by, at least, one miRNA [43]. Several studies have demonstrated the involvement of miRNAs in different biological processes such as proliferation, cell cycle regulation, proliferation, apoptosis, differentiation, development, metabolism, neuronal patterning, and aging [43–53].

**Figure 2:**
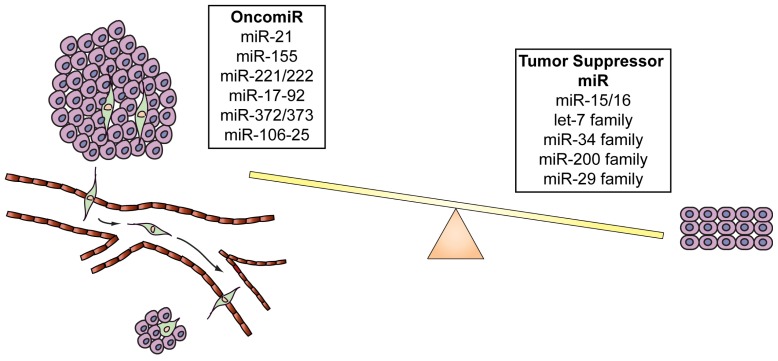
OncomiRs and tumor suppressor miRs. Correct cellular homeostasis is driven by a proper balance between oncomiRs and tumor suppressor miRs. OncomiRs are usually located in the amplified regions of the genome and are frequently over-expressed in cancer, promoting tumor growth and metastasis. Tumor suppressor miRs are often down-regulated in cancer and inhibit tumor growth inducing apoptosis and blocking cell migration.

MiRNAs are transcribed by an RNA polymerase II as long, capped and polyadenylated precursors called pri-miRNAs [54,55], which are further cleaved into hairpin-shaped ~70–100 nucleotides precursors (pre-miRNAs) by a ribonuclease III (Drosha) and the double-stranded DNA binding protein DGCR8/Pasha [56] (Figure 1). Exportin 5 then translocates the pre-miRNAs to the cytoplasm [57], where another RNAse III, Dicer [43,58] further processes the precursor in a double strand RNA of about 24 nt. The double-stranded RNA is incorporated into the RISC (RNA-induced silencing complex) but only one strand, the mature microRNA, remains stably associated with the RISC and will drive the complex to the target mRNA. If the base-pairing between miRNA and the 3′ UTR of the target mRNA is perfect the messenger is cleaved and degraded, whereas imperfect complementarity will result in translational silencing without mRNA degradation [59,60]. Several studies have demonstrated that miRNAs have a crucial role in cancer formation and spread. These small non-coding RNAs are, in fact, usually located in minimal regions of amplification, loss of heterozygosity, fragile sites, and common breakpoint regions in or in proximity of oncogenes or tumor suppressor genes. Moreover, profiling studies have demonstrated that miRNAs are differentially expressed in tumors *vs.* normal human tissues. These data have allowed the classification of microRNAs into two groups: oncomiRs (which act as oncogenes and are usually overexpressed in cancer, promoting tumor formation and spread) and tumor-suppressor miRs (which impair tumor growth and are usually silenced because of mutations, promoter methylation, or chromosomal rearrangements) [61–64], although some microRNAs can act as both oncogene or tumor-suppressor gene depending on the cellular context [65] (Figure 2 and Table 1).

### 3.1. OncomiRs

One of the most well-known oncomiRs is *miR-21*, overexpressed in different types of cancer such as chronic lymphocytic leukemia (CLL) [62], acute myelogenous leukemia (AML) [66], glioblastoma [67], pancreatic, prostate, colon, gastric, breast, and lung cancer [68]. In 2008, Asangani and coworkers [69] demonstrated that *miR-21* downregulated the tumor suppressor PCDC4 (programmed cell death 4) promoting tumor invasion and metastasis in colorectal cancer. Zhang and colleagues [70] showed that *miR-21* induced growth and invasion in non-small cell lung cancer by repressing PTEN (phosphatase and tensin homolog); moreover, *miR-21* modulate TRAIL sensitivity in glioma cells mainly by modulating caspase-3 and TAp63 expression and TRAIL-induced caspase machinery [71], confirming that *miR-21* acts like an oncogene by blocking the expression of critical apoptosis-related genes.

Another example of oncomiR is represented by *miR-155*. Similarly to *miR-21*, *miR-155* is highly expressed in CLL [72], AML [73], lung, breast and pancreatic cancer [68], Hodgkin disease [72], and primary mediastinal non-Hodgkin’s lymphoma [62]. In 2010, Jiang and coworkers demonstrated that *miR-155* targeted the tumor suppressor gene *Socs1* (*suppressor of cytokine signaling 1* gene) in human breast cancer cells, promoting cell proliferation, colony formation, and xenograft tumor growth [74]. *MiR-155* has also been found to be one of the most potent miRNAs suppressing apoptosis in human T cell leukemia (Jurkat cells) and in MDA-MB-453 breast cancer cells [75]. Moreover, in a transgenic mouse model, selective overexpression of *miR-155* in B cells led to early B cells polyclonal proliferation with a high-grade lymphoma-pre-B leukemia, suggesting that *miR-155* promotes the initiation and progression of the disease [76].

*MiR-221* and *-222* are also up-regulated in several solid tumors, such as hepatocarcinoma [77], breast estrogen negative cells [78], melanoma cells [79], thyroid cancer [80]. Both these miRNAs induce tumor growth and spread of several cancer cell lines [81–83]. In 2009, our group demonstrated that hepatocyte growth factor receptor (MET) oncogene, through Jun transcriptional activation, upregulated *miR-221* and -*222* expression, which in turn, by targeting *PTEN* and *TIMP3*, conferred resistance to tumor necrosis factor-related apoptosis-inducing ligand (TRAIL)-induced cell death and enhanced tumorigenicity of lung and liver cancer cells. Therefore, the use of microRNAs in therapeutic intervention could sensitize tumor cells to drug-inducing apoptosis and also inhibit their survival, proliferation, and invasive abilities [84].

The oncomiR group is wide, and comprises other microRNAs such as the *miR-17-92* cluster, which is crucial for B-cell proliferation and its absence induces an increase of the proapoptotic protein Bim and inhibits the pro-B to pre-B cell development [85]; *miR-372/373*, which are involved in the development of human testicular germ cell tumors by neutralizing the TP53 pathway [86]; *miR-10b*, which promotes cell migration and invasion in breast cancer [87]; the polycistron *miR-106-25*, which acts as an oncogene by interfering with the synthesis of p21 and Bim [88].

### 3.2. Tumor Suppressor MicroRNAs

The group of miRNAs able to inhibit cell growth, induce apoptosis, and block cell cycle, are called tumor suppressor miRs. Normally, oncomiRs are located mainly in the amplified regions in human cancers and are frequently over-expressed in neoplastic tissues. Conversely, tumor suppressor miRs are located in the deleted regions and are often down-regulated in cancerous tissues.

The first evidence that miRNAs are involved in cancer comes from the finding that *miR-15* and *miR-16* are down-regulated or deleted in most patients with chronic lymphocytic leukemia [61].

Their expression is inversely related to several oncogenes, such as *Bcl-2* [89], *CCND1*, *WNT3A* [90], *Ccne1*, *Bmi-1* [91], and *VEGF-A* [92], which induce cell proliferation, survival, invasion and angiogenesis. Recently it has been shown that *miR-15* and *-16* are involved in drug resistance. Pouliot *et al.* demonstrated that *miR-15* and *-16* sensitized cisplatin-resistant epidermoid carcinoma cells to apoptosis by targeting *WEE1* and *CHK1* [93].

Another example of tumor suppressor miR is represented by the *let-7* family. Several studies described the down-regulation of *let-7* family in numerous tumors, including lung [94], gastric [95], colon cancer [96], and Burkitt’s lymphoma [97]. *Let-7* family targets and inhibits the expression of several oncogenes such as *c-Myc* [97], *Ras* [98], *high-mobility group A* (*HMGA*) [99], *Janus protein tyrosine kinase* (*JAK*) and signal transducer and activator of transcription 3 (*STAT3*) pathway [100]. A recent study also reported that let-7 directly targets *PAK1*, *DIAPH2*, *RDX*, and *ITGB8*, multiple genes involved in the actin cytoskeleton pathway, inhibiting breast cancer cell migration [101].

The tumor suppressor activity of miR-34 family has been demonstrated in cancer cell types of lung [102], liver [103], breast [104], colon [105], brain [106], ovary [107], esophagus [108], and the lymphoid system [109]. In mammals, *miR-34* family comprises three processed miRNAs that are encoded by two different genes: *miR-34a* is encoded by its own transcript, whereas *miR-34b* and *-34c* share a common primary transcript. Their expression is directly induced by p53 in response to DNA damage or oncogenic stress [110]. *MiR-34* family inhibits many different oncogenic pathways involved in the control of cellular proliferation, cell cycle, and senescence by targeting oncogenes such as mitogen-activated protein kinase kinase 1 (*MEK1, MAP2K1*), R-Ras (*RRAS*), platelet-derived growth factor receptors (*PDGFRA and PDGFRB*) [111], and hepatocyte growth factor receptor (*MET*), *BCL2* and survivin.

*MiR-200* family is commonly lost in aggressive tumors such as lung, prostate and pancreatic cancer. It has been shown that *miR-200* family members directly target *ZEB1* and *ZEB2*, transcriptional repressors of E-cadherin [112], and *BMI1*, reducing epithelial mesenchimal transition [113].

*MiR-29s* are also downregulated in multiple cancer types such as CLL [62], breast [114] and cervical cancer [115], and hepatocellular carcinoma [116]. *MiR-29* family targets and inhibits *DNMT3A* and *-3B* (DNA methyltransferases 3A and 3B) [117], *Tcl1* in chronic lymphocytic leukaemia [118] and *Mcl1* in cholangio-carcinoma [119]. Moreover, it has been demonstrated that the down-regulation of *miR-29* by MYC, HDAC, and EZH2 promotes cell survival and growth in MYC-associated lymphomas [120]. In conclusion, the correct cell homeostasis and survival are driven by a proper balance between oncomiRs and tumor suppressor miRs. Up-regulation of oncomiRs or down-regulation of tumor suppressor miRs leads to cancer formation and progression (Figure 2 and Table 1).

## 4. snoRNAs

Small nucleolar RNAs (snoRNAs) are small non-coding RNAs whose length ranges from 60 to 300 nucleotides. SnoRNAs are normally located within introns of protein-coding genes and are transcribed by RNA polymerase II, but in some cases they can be found within introns of lncRNAs [121,122]. Within the cell, snoRNAs specifically accumulate in the nucleolar compartment, where they are responsible of the 2′-*O*-ribose methylation and pseudouridylation of specific ribosomal RNA nucleotides, essential modifications for the efficient and accurate production of the ribosome [123].

SnoRNAs can be classified into two groups: H/ACA box and C/D box. In both cases, snoRNAs hybridize specifically to the complementary sequence in the rRNAs and the associated protein complexes (C/D or H/ACA snoRNP) carry out the appropriate modification on the nucleotide that is identified by snoRNAs [124–126].

The H/ACA box snoRNAs family is involved in pseudouridylation of rRNAs. These ncRNAs have two major hairpin elements, connected by a hinge, and followed by a short tail region containing the conserved H and ACA box motifs that are located at the bases of the 5′ and 3′, respectively. The sequence specificity for the pseudouridylation is carried by two short antisense elements located in an internal loop of the 5′ and/or 3′ hairpins [127].

**Figure 3:**
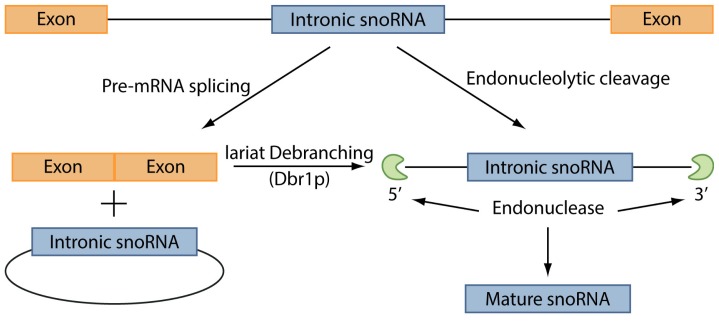
Intronic snoRNA processing. SnoRNA maturation occurs through two distinct pathways: splicing-dependent and splicing-independent. In the first pathway, the splicing of a pre-mRNA leads to a snoRNA-containing lariat, which is linearized by the enzyme Dbr1p and then endonucleases and exonucleases release the mature snoRNA. In the splicing-independent pathway the snoRNA is directly excided from the intron of the pre-mRNA by endonucleolytic cleavage.

The C/D box snoRNAs, instead, are mainly involved in the 2′-*O*-methylation of rRNAs. This group of ncRNAs contains two short sequence motifs, box C (5′-PuUGAUGA-3′) and box D (5′-CUGA-3′) located near the 5′ and the 3′ ends, respectively. These elements form a terminal stem-box structure, involving not only elements required for snoRNAs nuclear localization, but also another copy of the box C, named box C′, in their central portion, and another box D, named box D′. 2′-*O*-methylation is carried out through one or two antisense elements located upstream box D and/or box D′ and complementary to a site of rRNA 2′-*O*-ribose methylation [128]. The process of snoRNAs maturation has not been entirely unveiled, however it has been demonstrated that the maturation of box C/D snoRNAs in yeast can occur through two pathways (Figure 3) [129]. In the first pathway, splicing of a pre-mRNA leads to a snoRNA-containing lariat, which is then linearized by the enzyme Dbr1p. Thanks to the activity of endonucleases and exonucleases the mature snoRNA is finally released. The second pathway, instead, is splicing-independent: the snoRNA is excided from the intron of the pre-mRNA directly, leading to the destruction of the mRNA. However, this latter pathway is still not well defined and the enzymes involved in this process have not been totally identified.

Although the main function of snoRNAs seems to be related to rRNA folding and stabilization, recent discoveries have pointed out a wider regulatory function for these small ncRNAs. For example, snoRNAs seem to be involved in miRNA synthesis. In 2010, Breimer and coworkers identified several box C/D sno-miRNAs, originating from relatively short snoRNAs (such as *U27* and *HBII-336*) displaying miRNA features in mRNAs silencing in different cell types, therefore controlling several biological processes normally regulated by miRNAs [130].

SnoRNAs are also involved in the onset of the Prader-Willy syndrome (PWS), induced by the genetic loss of the 15q11–q13 locus, normally active only on the paternal allele. This site is characterized by several copies of the *HBII-85* snoRNA, whose loss seems to be correlated with the PWS phenotype, both in human and in mice [131,132]. Moreover, recent studies reported the involvement of snoRNAs in cancer formation and progression, although the exact molecular mechanisms by which snoRNAs regulate cancer are still unknown.

Similarly to miRNAs, snoRNA expression has been found deregulated in cancer patient samples. In fact, the expression of *GAS5* (growth arrest specific 5), a gene which encodes an lncRNA but also harbors ten intronic snoRNAs, is downregulated in breast cancer compared to normal adjacent epithelial breast tissue. *GAS5* transcript sensitizes mammalian cells to apoptosis inducers, therefore displaying a tumor-suppressor role [133]. Moreover, Nakamura and coworkers demonstrated that *GAS5* was a partner of BCL6 in a patient with diffuse large B-cell lymphoma, carrying the chromosomal translocation t (1; 3) (q25; q27) [134], while Gee showed that *GAS5* low expression correlated with poor prognosis in breast cancer and head and neck squamous carcinoma [135]. The same authors also reported that snoRNA U50 is frequently transcriptionally downregulated in breast and prostate cancer [136] and that its 2-nucleotides somatic and germline deletion led to increased incidence of homozygosity for the deletion in cancer cells.

Other snoRNAs, such as *snoRNA42*, overexpressed in NSCLC cells, are located at frequently amplified genomic regions in tumors, therefore acting like oncogenes and promoting tumor growth. In 2011 Mei and coworkers found that *snoRNA42* knockdown in NSCLC cells impaired tumorigenicity *in vitro* and *in vivo* promoting apoptosis in a p53-dependent manner; conversely its enforced expression in bronchial epitheliums promoted cell growth [137].

Moreover, Liao *et al.* performed a profiling study on 22 NSCLC tissues. They found an overexpression of six snoRNAs compared to normal specimens. In addition to *snoRNA42*, they identified *SNORD33*, *SNORD66*, *SNORD73B*, *SNORD76* and *SNORD78*. Of these, *SNORD33*, *SNORD66*, and *SNORD76* expression in the plasma of NSCLC patients was higher compared to cancer-free individuals [138]. It has been demonstrated that, in addition to deregulated snoRNAs, also mutations of genes encoding for snoRNPs (snoRNA-associated proteins) can promote tumorigenesis. One of these genes is the human dyskerin, a putative pseudouridine synthase involved in the rRNA pseudouridylation and in the stabilization of the telomerase RNA elements. Mutations of its gene, *DKC1*, cause the X-linked genetic disease dyskeratosis congenita and promote tumor formation in mice [139]. The same effects have been described when point mutations occur in the genes encoding NOP10 and NHP2, both components of the H/ACA snoRNPs.

## 5. Long Noncoding RNAs

Several studies based on RNA deep sequencing and genome-wide analysis have recently pointed out that the genome of mammals, as well of other organisms, contains thousands of long transcripts whose length ranges from 200 nt to 100 kilobases, called long non-coding RNAs (lncRNAs or lincRNA, for long intergenic ncRNA) [3,140–144]. LncRNAs are located within nuclear or cytosolic fractions [145]. They are usually transcribed by RNA polymerase II but have no open reading frame [146], and map to intronic and intergenic regions [147]. Moreover, they display epigenetic features common to protein-coding genes, such as trimethylation of histone 3 lysine 4 (H3K4me3) at the transcriptional start site (TSS), and trimethylation of histone 3 lysine 36 (H3K36me3) throughout the gene body [148,149]. It has been estimated that nearly 15,000 lncRNAs are present in the human genome, but only a small fraction is expressed in a given cell type. All the information regarding identified lncRNAs has been catalogued and is available at the website http://www.lncrnadb.org [150–152].

Although they were initially thought to be the product of a “noisy” inconsequential transcription resulting from low RNA polymerase fidelity [142], recent studies have demonstrated that lncRNAs regulate several biological processes such as transcription [153–156], translation [157], cellular differentiation [158], regulation of gene expression [159], cell cycle regulation [160,161], chromatin modification [143,162,163], and nuclear-cytoplasmic trafficking [159,164–167].

LncRNAs have been also found to guide protein complexes which regulate chromatin modification or transcription to their targets [143,162,168,169]. Finally, it has been demonstrated that lncRNAs are dysregulated in several human diseases, including cancer.

Dysregulated expression of lncRNAs in cancer marks the spectrum of disease progression [170] and may serve as an independent predictor for patient outcomes [171].

Long non-coding RNAs can mediate epigenetic changes by recruiting chromatin-remodeling complexes to specific genomic loci. A recent study found that 20% of 3300 human long non coding RNAs are bound by Polycomb Repressive Complex 2 (PRC2) [162]. Although the specific molecular mechanisms are not defined, there are several examples that illustrate the silencing potential of lncRNAs. The first, most known example is represented by *Xist* (X-inactive-specific transcript) gene, which encodes an lncRNA crucial for the inactivation of the X-chromosome in mammals [172]. Basically, *Xist* physically coats one of the two X-chromosomes and recruits the chromatin regulator PRC2 (Polycomb chromatin remodeling complex) to this chromosome, promoting the formation of heterochromatin through histone modifications [173]. Another important example is represented by the hundreds of long ncRNAs which are sequentially expressed in the human homeobox (Hox) loci, where they define chromatin domains of differential histone methylation and RNA polymerase accessibility [174]. One of these ncRNAs, Hox transcript antisense RNA (*HOTAIR*) regulates *in trans* human *HOXD* genes expression through the induction of a repressive chromatin state. This occurs through the association of *HOTAIR* with the chromatin-modifying complexes PRC2, LSD1, and coREST/REST [143,162,171]. As modulator of epigenetic landmark, it has been shown that *HOTAIR* has a profound effect on tumorigenesis. Indeed, it is upregulated in breast and colon cancers and it is associated with metastasis and poor prognosis [171]. Another important effect of lncRNAs on chromatin modification with important consequences in cancer is represented by the lncRNA *ANRIL*, which controls the epigenetic status of the locus *INK4b/ARF/INK4a* by interacting with subunits of PRC1 and PRC2. High expression of *ANRIL* has been found in some cancer tissues such as melanoma and prostate cancers ([175,176]. The long noncoding RNA *MALAT1* (metastasis-associated lung adenocarcinoma transcript 1), also known as *NEAT2* (nuclear-enriched abundant transcript 2), is a highly conserved nuclear noncoding RNA (ncRNA) which acts as molecular decoy serving as a structural link in ribonucleoprotein (RNPs) complexes. Gutschner and colleagues developed a *MALAT1* knockout model in human lung tumor cells. In lung cancer, *MALAT1* does not alter alternative splicing but actively regulates gene expression including a set of metastasis-associated genes. Consequently, *MALAT1*-deficient cells are impaired in migration and form fewer tumor nodules in a mouse xenograft. Antisense oligonucleotides (ASO), blocking *MALAT1*, prevent metastasis formation after tumor implantation [177]. In addition to these active lncRNAs acting as oncogenes, there are also lncRNAs with tumor suppressor function. One very famous example is the ncRNA *GAS5* (Growth Arrest-Specific 5). It was originally identified based on its increased levels in growth-arrested mouse NIH3T3 fibroblasts [132]. *GAS5* binds to the DNA-binding domain of the glucocorticoid receptor (GR) by acting as a decoy glucocorticoid response element (GRE), thus competing with DNA GREs for binding to the GR [156]. *GAS5* negatively regulates the survival of lymphoid and breast cells, and is aberrantly expressed in several cancers. Pickard *et al.* showed that *GAS5* promotes apoptosis of prostate cells after irradiation with UV-C light and low levels of *GAS5* expression may therefore reduce the effectiveness of chemotherapeutic agents [178].

Recently, lncRNAs have also shown their tumorigenic potential by modulating the transcriptional program of p53 [179].

A 3kb lncRNA, *linc-RNA-p21*, transcriptionally activated by p53, has been shown to collaborate with p53 in order to control gene expression in response to DNA damage. Silencing of *lincRNA-p21* derepresses the expression of hundreds of genes through the interaction with hnRNP-K (Heterogeneous nuclear ribonucleoprotein K), thus, promoting apoptosis of abnormal cells or restraining tumors [179].

LncRNA *PANDA* is induced in response to external stimuli in a p53-dependent manner. After DNA damage p53 directly binds to the *CDKN1A* locus and activates *PANDA*, which enables cell-cycle arrest and impairs the expression of pro-apoptotic genes thanks to its interaction with the transcription factor NF-YA [LNC8] [180].

In addition to the features described above, recent studies have unveiled other properties of lncRNAs. For instance, it has been demonstrated that pseudogene transcripts are biologically active as they can regulate mRNA stability. One example is given by the tumor suppressor pseudogene *PTENP1*, whose 3′ UTR region is very similar to the untraslated region of *PTEN* transcript. Both these regions bind the same set of miRNAs, and *PTENP1* pseudogene may act as “decoy” by protecting *PTEN* mRNA from common miRNA binding and allowing the expression of the tumor suppressor protein. *PTENP1* pseudogene therefore belongs to the group of competing endogenous RNAs (ceRNAs). Similarly, *KRAS* and *KRAS1P* transcript levels have been found positively correlated, corroborating that pseudogene functions mirror the role of their cognate genes as explained by a miRNA decoy mechanism. In cancer, specific mutations at the binding site of these pseudogenes impair their activity, therefore promoting tumor progression [181].

Enhancer-like lncRNAs (eRNAs) were discovered by Ørom and colleagues in 2010 [141]. The authors used a GENCODE annotation of the human genome to characterize over a thousand lncRNAs in several cell lines, finding that some of these RNAs displayed an enhancer-like function. Depletion of these ncRNAs led to a decreased expression of their neighboring protein-codon genes, such as the regulator of hematopoiesis *SCL*, *Snai1*, and *Snai2*, indicating that eRNAs play a pivotal role in development and differentiation. Moreover, Melo *et al.* showed through genome-wide chromatin-binding profiles that p53 protein binds also to regions located at distant sites from any known p53 target gene. Such regions were characterized not only by conserved p53-binding sites but displayed also enhancer activity and interacted with multiple neighboring genes allowing long-distance p53-dependent transcription regulation [182].

Finally, Natural Antisense Transcripts (NATs) are a large class of lncRNA transcribed from the opposite DNA strand to other transcripts and overlap in part with sense RNA. NATs play an important role in antisense regulation in gene expression. NATs have been implicated in several processes such as RNA translation [183] and transcriptional interference [184], and they have a pivotal role also in cancer. *aHIF*, a NAT derived from the 3′ UTR of *HIF1*, represents the first case of overexpression of a NAT associated with a specific human malignant disease: non-papillary clear-cell renal carcinomas, but not in papillary renal carcinomas [185]. Moreover, it has been demonstrated that *aHIF* expression is a poor prognosis marker in breast cancer [186].

The already mentioned *ANRIL* is an antisense lncRNA originates from the *INK4B-ARF-INK4A* locus, which contains three tumour suppressor genes, and it is overexpressed in prostate cancer tissues. Repression of *ANRIL* expression was associated with a reduction in cellular proliferation and increased the expression of both p16^Ink4A^ and p15^INK4B^, which are encoded by *CDKN2A* and *CDKN2B*, respectively [176]. *BOKAS* is a natural antisense transcript of Bok, a proapoptotic member in the Bcl-2 family. The expression of *BOKAS* was only detected in testis and different cancer tissues but not in other normal adult tissues. Overexpression of *BOKAS* was able to inhibit Bok-induced apoptosis in HeLa cells [187].

Another example of NAT is represented by *Zeb2/Sip1* NAT. This NAT regulates E-cadherin expression by increasing the levels of Zeb2 protein, a transcriptional repressor of E-cadherin, suggesting a role for noncoding RNAs in the control of epithelial morphology [188].

## 6. Conclusions

The recent discoveries regarding the biogenesis and function of ncRNAs have definitely improved our undestanding of the complexity of the human genome and the regulation of several processes. In particular, the involvement of microRNAs in the regulation of cell cycle, proliferation, differentiation and, most of all, in cancer formation and progression has certainly opened new fields of research aimed to better elucidate their mechanisms of action. Recently, our group reported that miRNAs secreted through exosomes bind to Toll-like receptor 8 (TLR8) in human and TLR7 in mouse inducing a pro-inflammatory response [189]. Therefore, in addition to their post-transcriptional regulatory function, miRNAs act like hormones and are involved into cell-to-cell communication. Other studies have shown the presence of tumor-derived microRNAs in serum or plasma as an approach for blood-based detection of human cancers, indicating that microRNAs could be used as circulating biomarkers [190]. Several groups are currently investigating the possibility to use microRNAs as therapeutic tools alone or in combination with chemotherapy.

Successfull systemic delivery of miRNAs as anti-cancer approaches in preclinical models using liposomes [191], viral vectors [192], and nanoparticles [193] has been reported. There is no doubt that miRNAs and other ncRNAs play a very important role in the regulation of pathways involved in tumor development and progression. Although there are still several obstacles to overcome before clinical testing of miRNA therapeutics, such as delivery and chemical modification of miRNA modulators, the fact that ncRNAs are natural antisense interactors and regulate many genes involved in survival and proliferation makes them excellent candidates to become powerful therapeutic tools in the near future.

## Figures and Tables

**Table 1:** OncomiRs and tumor suppressor miRs.

miRNA	Tumor type	Status	References
*miR-21*	CLL, AML, glioblastoma, pancreatic, prostate, colon, gastric, breast and lung cancer	Up-regulated	[62,66–71]
*miR-155*	CLL, AML, lung, breast and pancreatic cancer, Hodgkin disease, primary mediastinal non-Hodgkin’s lymphoma	Up-regulated	[62,66,68,72,74–76]
*miR-221/222*	hepatocarcinoma, breast cancer, melanoma, thyroid cancer and glioma	Up-regulated	[77–84]
*miR-17-92*	AML	Up-regulated	[85]
*miR-372/373*	testicular germ cell tumor	Up-regulated	[86]
*miR-10b*	breast cancer	Up-regulated	[87]
*miR-106-25*	gastric cancer	Up-regulated	[88]
*miR-15-16*	CLL, prostate and ovarian cancer and multiple myeloma	Down-regulated	[61,89–93]
*let-7* family	lung, gastric, colon, breast cancer and Burkitt’s lymphoma	Down-regulated	[94–101]
*miR-34*	lung, liver, breast, colon, brain, ovary, esophageal cancer and non-small cell lung cancer (NSCLC)	Down-regulated	[102–111]
*miR-200*	lung, prostate and pancreatic cancer	Down-regulated	[112,113]
*miR-29*	CLL, breast and cervical cancer hepatocellular and cholangio-carcinoma	Down-regulated	[62,114–120]
